# Cancer exosomes and natural killer cells dysfunction: biological roles, clinical significance and implications for immunotherapy

**DOI:** 10.1186/s12943-021-01492-7

**Published:** 2022-01-14

**Authors:** Reza Hosseini, Hamzeh Sarvnaz, Maedeh Arabpour, Samira Molaei Ramshe, Leila Asef-Kabiri, Hassan Yousefi, Mohammad Esmaeil Akbari, Nahid Eskandari

**Affiliations:** 1 Department of Immunology School of Medicine, Isfahan University of Medical Sciences, Isfahan, Iran; 2 Department of Immunology School of Public Health, Tehran University of Medical Sciences, Tehran, Iran; 3 Department of Medical Genetics School of Medicine, Tehran University of Medical Sciences, Tehran, Iran; 4 Student Research Committee, Department of Medical Genetics, School of Medicine Shahid, Beheshti University of Medical Sciences, Tehran, Iran; 5 Surgical Oncologist Cancer Research Center, Shahid Beheshti University of Medical Sciences, Tehran, Iran; 6 Department of Biochemistry and Molecular Biology, LSUHSC School of Medicine, New Orleans, USA

## Abstract

Tumor-derived exosomes (TDEs) play pivotal roles in several aspects of cancer biology. It is now evident that TDEs also favor tumor growth by negatively affecting anti-tumor immunity. As important sentinels of immune surveillance system, natural killer (NK) cells can recognize malignant cells very early and counteract the tumor development and metastasis without a need for additional activation. Based on this rationale, adoptive transfer of ex vivo expanded NK cells/NK cell lines, such as NK-92 cells, has attracted great attention and is widely studied as a promising immunotherapy for cancer treatment. However, by exploiting various strategies, including secretion of exosomes, cancer cells are able to subvert NK cell responses. This paper reviews the roles of TDEs in cancer-induced NK cells impairments with mechanistic insights. The clinical significance and potential approaches to nullify the effects of TDEs on NK cells in cancer immunotherapy are also discussed.

## Introduction

Cancer cells actively release a variety of soluble biomolecules such as cytokines, chemokines, and growth factors to establish tumor microenvironment [1–4]. Over the last decades, extracellular vesicles, especially exosomes, have been known as an important tool of cancer cells in communicating with stromal and distant cells [5–7]. Emerging evidence shows that tumor-derived exosomes (TDEs) contain a variety of molecular components ranging from lipids, membrane-associated proteins, long non-coding RNAs (lncRNAs) and microRNAs (miRNAs) which can alter the behaviour of recipient cells and provide contact-independent routes for the growth of malignant cells [8, 9]. It is increasingly becoming clear that tumor exosomes are involved in several processes of tumor formation and invasion, including angiogenesis, proliferation and growth, metastasis and immune escape [9–11].

Of particular note, several lines of evidence support that TDEs are the key immunomodulatory players of tumor microenvironment [12]. In this regard, these tumor-derived particles were shown to manipulate both innate and adaptive immune responses in favor of tumor progression [12, 13]. Numerous studies have shown that exosomes derived from tumor cells demolish anti-tumor immunity by impairing the function of DCs, NK cells and T cells [14, 15]. Among them, NK cells are considered as initial responders to malignant cellular transformation. Despite the CD8 + CTL responses, NK cells do not require prior antigen exposure to recognize tumor cells, marks them as the early line of defense against cancer cells [16]. Indeed, NK cells express a repertoire of inhibitory (KIR, CD94-NKG2A, etc.) and activating receptors (e.g. NKG2D, NKp30, NKp44, and NKp46) which defines their fate and enable them to recognize their ligands on transformed cells [17]. Although lower expression of the major histocompatibility complex I (MHCI) molecules and ligands for activating receptors on tumor cells is assumed to stimulate NK cells activity, but tumor cells could dampen NK cells function via different mechanisms [16, 18]. Recent data have pointed out that tumor exosomes play critical roles in NK cells dysfunction [19–21]. It is believed that TDEs can be taken up by NK cells or induce downstream signals through receptor-ligand interactions, downmodulating their anti-tumor activity [13, 22]. There is also a large body of evidence indicating that tumor exosomes harbor a plethora of surface ligands and biomolecules that can interfere with the recruitment, cytokine production, molecular expression and cytolytic activity of NK cells [23]. Notably, TDEs are also assumed to counteract the beneficial effects of NK-based immunotherapy [14, 23]. Although some reports have shown an immunostimulating role of cancer-derived extracellular vesicles [24–31], but here we will mainly focus on their inhibitory effects and review the roles of TDEs in NK-cells dysfunction with mechanistic insights and summarize the clinical significance and therapeutic approaches to counter exosome-dependent tumor-induced NK cells impairment in treating cancers.

### Uptake/Interactions of TDEs with NK cells

**Fig. 1:**
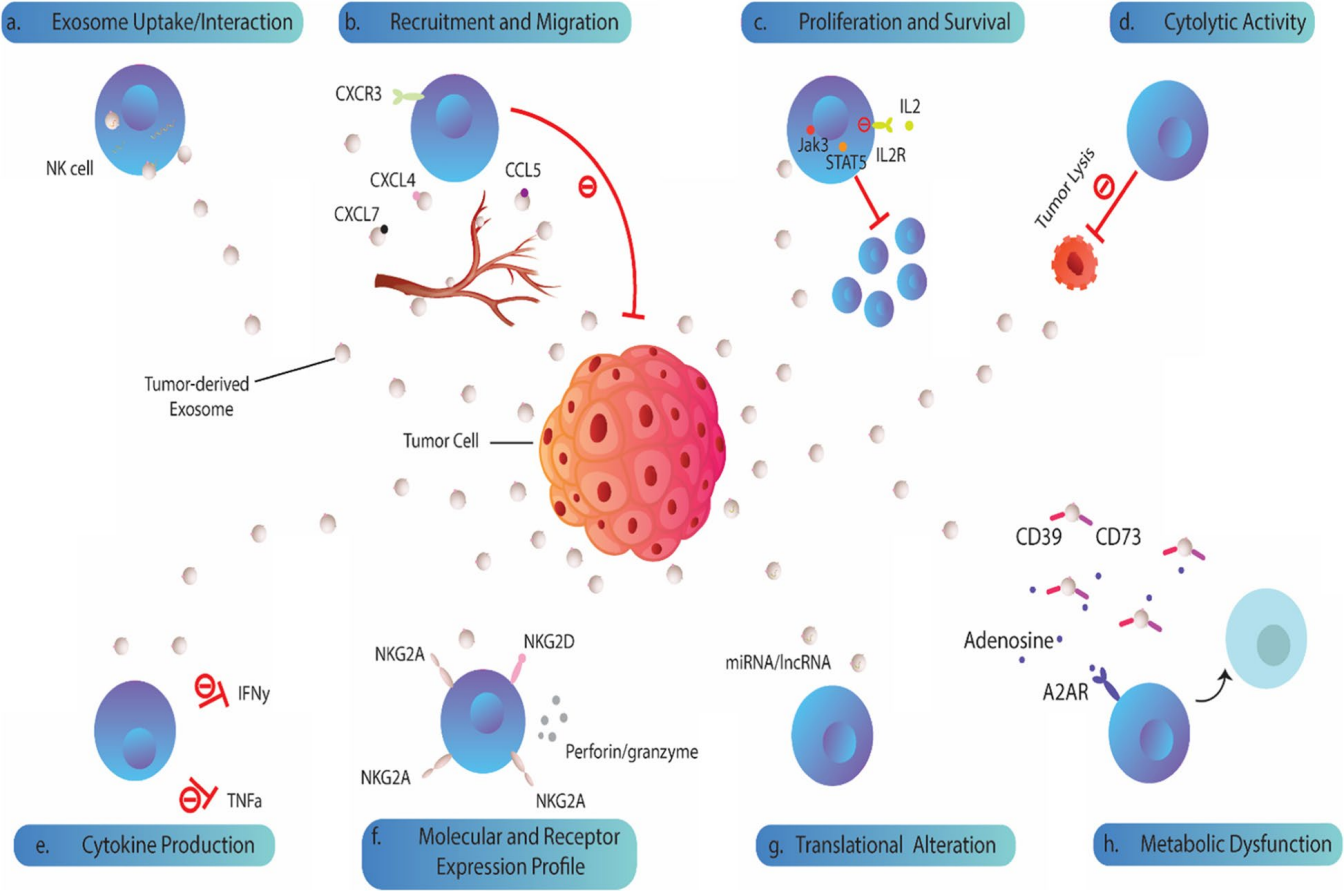
Biological impacts of tumor-derived exosomes on NK cells. **a**) Tumor-derived exosomes (TDEs) can be taken up by or interact with human NK cells. **b)** By expressing surface chemokine/chemokine receptors, TDEs can inhibit recruitment and migration of NK cells toward tumor milieu. **c)** These virus-sized particles can also block the effect of IL-2 on NK cells proliferation in a concentration dependent manner, either via decreasing the phosphorylation of JAK3 and STAT-5 or downmodulating IL-2R on NK cells. **d)** Cytolytic activity, and **e)** secretion of key cytokines, IFN-γ and TNF-α, are also compromised in exosome-exposed NK cells. **f)** Tumor exosomes can downmodulate the expression of activating receptors on NK cells, such as NKG2D, and cytotoxic mediators, including perforin and granzyme, demolishing tumor recognition and lysis by NK cells. **g)** miRNA (miR-92b and miR-23a) or lncRNA (circUHRF1 and SNHG10) cargo of TDEs may alter translational status of cells, leading to NK cell suppression. **h)** Co-expression of CD39/CD73 pair on TDEs drive higher levels of adenosine production that can be engaged with cognate A2AR receptors on NK cells, leading to their metabolic dysfunction

Tumor-derived exosomes (TDEs) can be taken up by various cells, preferentially immune cells, through plasma membrane fusion, endocytosis, phagocytosis, micro pinocytosis, and lipid raft-mediated internalization [14, 32]. The uptake/interaction of tumor exosomes with immune cells is believed to participate in immune suppression and tumor escape [32, 33]. There are multiple findings indicating that tumor exosomes can deliver their cargo into NK cells via fusion with the cell membrane, hindering their anti-tumor function [19]. Studies have shown that exosomes from pancreatic cancer cells (L3.6pl) and murine mammary carcinoma cells (TS/A) are taken up by NK cells and stably present in cytoplasm which is accounted for their decreased cytotoxic activity [34, 35]. Oral cancer-derived exosomes and those obtained from leukemic cell line (Jurkat cells) were also demonstrated to be internalized by NK and NK-92MI cells [22, 36]. In vivo experiments also showed that the inject exosomes derived from gastric cancer cell lines (MKN-45, MKN-28, and SGC-7901) are mainly taken up by NK cells and macrophages, contributing to the lung metastasis of gastric cancer cells [13]. In a study on lung cancer, microvesicles (MVs) derived from normoxic and hypoxic IGR-Heu and K562 tumor cells are also internalized by NK cells at the same levels [37]. However, contrary to the above-mentioned studies, NK cells exhibited poor capability in uptake of exosomes-derived from breast cancer (EO771) cells, lymphoma-derived exosomes and those isolated from MCL (mantle cell lymphoma) patients [32, 38, 39]. This is in agreement with the observations that the efficiency of uptake by NK cells differ markedly for the exosomes derived from different tumors (including hepatoblastoma (HepG2 cells), cervix cancer (Hela cells), breast cancer (MCF-7 cells), myeloid leukemia cells (K562), and T leukemia cells (Jurkat)) [40]. Indeed, there might be some exosomes-associated molecules determining their cellular uptake. For example, blocking of phosphatidylserine (PS) was found to inhibit the uptake of ovarian cancer-derived exosomes by NK cells, suggesting a PS-dependent uptake mechanism [41]. Apart from their uptake, several lines of evidence also show that TDEs may affect immune cells (T cells, macrophages, dendritic cells, Regulatory T cells) by triggering signals via ligand-receptor interactions [42–44]. In accordance, it was demonstrated that TDEs reprogram NK-92 cells to block their anti-leukemia cytotoxic functions, mainly through signaling via surface receptors [23]. The corresponding results showed that the interaction of exosomal ligands (including but not limited to TGF-b) with their cognate receptors on NK cells can induce multiple downstream inhibitory signaling pathways, suppressing their anti-tumor activity [23] (Fig. 1a).

### TDEs regulate recruitment and migration of NK cells

Shreds of evidence indicate that TDEs actively affect recruitment and migration of cancer and immune cells to establish tumor microenvironment and metastatic niche [42, 45, 46]. A previous study showed that tumor exosomes mediate the migration of MDSCs and contribute to the metastasis of murine breast cancer cells (4T1 cells) to the lung in a CCL2-dependent manner [47]. Others have shown that exosomes-derived from B16F10 (mouse melanoma; epithelial-like cells), CT26.WT (mouse colon carcinoma; fibroblasts), and LTPA (mouse pancreatic adenocarcinoma; epithelial cells) have high levels of CX3CR1 [48]. It was revealed that this exosomal CX3CR1 could bind to its soluble ligand (CX3CL1), suggesting that might play a role in intercellular crosstalk [48]. Exosomal chemokine from heat-stressed tumor cells were also shown to affect the mobility of DCs [49]. Besides, horizontal transfer of exosomal chemokine receptors, such as CXCR4, has been shown to enhance the migration of hepatocellular carcinoma cells [50]. Similar to these findings, a recent study demonstrated that exosomes isolated from AML patients could significantly reduce the migration of NK-92 cells toward tumor cells [23]. Further proteome profiling revealed that AML exosomes are highly enriched in CXCL4, CXCL7, and CCL5 (RANTES) compared to exosomes obtained from healthy donors [23]. Notably, the co-incubation with AML exosomes reduced CXCR3 expression on NK-92 cells surface, suggesting that down-regulation of CXCR3 levels by AML exosomes might be ligand-mediated and responsible for the decreased migration of NK-92 cells into the tumor site [23] (Fig. 1b). Such findings indicate that TDEs could impact several important biological processes, including chemotaxis and recruitment of immune cells (such as NK cells) into the tumor site.

### TDEs affect proliferation and survival of NK Cells

The literature supports that tumor secretome, including TDEs; dominantly favor tumor progression by downregulating proliferation and survival of immune cells [51–53]. In a pioneer research, exploring the effects of tumor exosomes on the proliferation and survival of NK cells, it was revealed that pretreatment with murine mammary carcinoma (TS/A cells) exosomes reduces the number and percentage of NK cells in vitro [35]. Alongside, the injection of TS/A exosomes into Balb/C mice was also found to significantly reduce NK cells number and percentage in the lung, but not in the liver and lymph nodes. With attention to the details, the total number of splenocytes and NK cells was observed to be increased following the treatment of mice with tumor exosomes; however, the percentage of NK cells in the spleen was decreased [35]. These findings implied that TS/A-exosomes may inhibit NK cells proliferation in vivo, leading to the tumor immune-escape. To further elucidate this observation, researchers have examined the effect of tumor exosomes on IL-2-dependent NK cell proliferation pathway [35]. Subsequent results showed that not only TS/A exosomes but also exosomes from MDA231 (human breast cancer), A2058 (human melanoma), and the 4 T.1 (murine breast cancer) cell lines could significantly block the proliferation of NK cells induced by IL-2 [35]. More detailed investigation of signaling pathways downstream of IL-2R revealed that the activity of p42/p44 and of Akt, a substrate of PI3K, in NK cells did not change following the treatment with TDEs. However, exosomes were able to inhibit Jak3 expression, as a reduction in the levels of phosphorylated Stat5 was observed [35]. Of particular note, the resultant decrease in Stat5 phosphorylation was dependent on the concentration of TDEs (Fig. 1c). Similarly, a dose-dependent reduction in cyclin D3 and phosphorylated levels of its substrate Rb was also seen in NK cells treated with tumor exosomes. However, it was found that the reduced cytotoxic activity of NK cells treated with TS/A exosomes is not due to apoptosis, and co-incubation with TS/A exosomes did not change the viability of NK cells [35]. Exosomes from human tumor cells (mesothelioma cell line) was also found to selectively impair peripheral blood lymphocytes response to IL-2 [54]. However, it was noted that these exosomes selectively downmodulate expression of the high-affinity IL-2 receptor on cytotoxic effector cells including NK cells, thereby inhibit their IL-2 driven proliferation [54]. Other research have also shown a decreased proliferation of NK-92 incubated with AML exosomes [23] and a decreased frequency of CD8 + T and NK cells following the injection of gastric cancer-derived exosomes; however these anti-proliferative effects have not been attributed to the induction of apoptosis [13, 23]. Treating with multiple myeloma-derived exosomes (MM-EXs) also affected neither apoptosis nor proliferation of NK cells [55] (Fig. 1c).

### TDEs alter cytolytic activity of NK cells

In spite of inhibiting the early stage of tumor formation, NK cells also are able to eradicate solid tumors through cytotoxic functions [56, 57]. However, studies have confirmed that tumor cells robustly inhibit tumoricidal activity of NK cells. Recent data show that, in addition to reducing NK cells count, tumor exosomes are a candidate that reduces cytolytic activity of NK cells in tumor context [23]. It has been demonstrated that the treatment of NK-92 cells with AML exosomes significantly inhibits their cytotoxicity against K562 cells [23]. Likewise, NK cells pre-treated with pancreatic cancer-derived EVs exhibited decreased cytotoxicity against pancreatic CSCs [34]. Such a mechanism is assumed may help CSCs to avoid of elimination by NK cells, potentially leading to tumor recurrence [58, 59]. It is interesting to note that saliva exosomes derived from pancreatic cancer cells were also shown to decrease cytolytic potential of NK cells both in vitro and in vivo [60]. Others investigating the effect of oral cancer-derived exosomes on the cytotoxicity of NK cells have reported that the oral cancer cell-killing effect of NK cells could be increased following the co-incubation of NK cells with the OCEXs in a short time, however by increasing the incubation time, the cytotoxicity of NK cells was dramatically decreased [22]. These findings imply that although TDEs may stimulate NK cells cytotoxicity at short time, but long time exposure to TDEs can inhibit their cytolytic function, resulting in immune escape and cancer progression. Similarly, it was also shown that NK cells cytotoxicity is impaired in mice treated intra-peritoneally with the exosomes either produced by murine mammary TS/A or 4 T.1 tumor cell lines [35]. Furthermore, although similar uptake patterns have been observed for normoxic and hypoxic tumor-derived MVs, but NK cells co-cultured with normoxic or hypoxic MVs displayed different levels of cytotoxicity [37]. Strikingly, the NK cells treated with hypoxic tumor-derived MVs exhibited significantly lower cytotoxicity toward IGR-Heu or K562 tumor cells than normoxic MVs-treated NK cells [37] (Fig. 1d).

Consistent with the previous studies, treatment with multiple myeloma exosomes was also shown to significantly reduce the cytotoxic function of NK cells against K562 cells [55]. Anti-tumor activity of CD3 − CD56 + NK cells against K562 targets was also significantly inhibited by exosomes isolated from plasma of HNC patients; while no suppression was observed with exosomes isolated either from healthy donors or those patients with no evident disease [23]. Others have revealed that exosomes of AML and head and neck cancer (HNC) patients as well as glioblastoma-derived exosomes (GBex) markedly suppress human NK cells function [61–63]. In a well-designed study, researcher have examined whether exosomes derived from clear cell renal cell carcinoma (ccRCC) cells are involved in the process of deactivation of NK cells [64]. For this purpose, purified human NK cells were cultured in a transwell coculture system with primary cells derived from non-tumor tissue (NT), margin region (M) and tumor (T) tissues separately. It was found that only exosomes from primary ccRCC cells (T) induce NK cell dysfunction. More importantly, the findings revealed that exosomes isolated from ccRCC cells in advanced stage (III/IV) had more suppressive effects on NK cells than those in early stage (I/II) [64] (Fig. 1d).

### TDEs modulate cytokine production by NK cells

Since TDEs exert large effects on immune cells, thus it would be expected that exposure to tumor exosomes can also alter cytokine production by NK cells [13, 42, 65]. Tumor necrosis factor-α (TNF-α) and interferon-γ (IFN-γ) are two main cytokines produced by activated NK cells, orchestrating anti-tumor immune responses [66, 67]. Exosomes from cholangiocarcinoma were found to significantly inhibit NK cells secretion of TNF-α, reducing their anti-tumor function [68]. Similarly, NK cells co-incubated with pancreatic cancer (L3.6pl)-derived EVs had a significant decrease in TNF-1, and IFN-γ production [34]. Treatment with TS/A (murine mammary carcinoma cells) exosomes were also shown to significantly inhibit the release of IFN-γ in IL-2-stimulated NK cells, suggesting that tumor exosomes could impair production of cytokines by activated NK cells [35]. Reduced IFNγ expression has also been observed in NK cells pre-treated with normoxic or hypoxic tumor-derived MVs [37]. Notably, hypoxic tumor MVs exhibited more significant effects on the decrease of IFNγ production by NK cells compared to normoxic MVs, and decreased cytotoxicity of exosome-treated NK-92/NKD cells was found to be directly correlated with the reduced expression of IFNγ by these cells [37] (Fig. 1e).

### TDEs alter receptor and molecular expression patterns of NK cells

The function of NK cells is known to be tightly regulated by activating and inhibitory receptors [69]. NKG2D and NKp30, NKp44, NKp46 are important activating receptors on NK cells and their expression levels determine the antitumor capacity of NK cells [70–72]. On the other side, the expression of NKG2A, one of the most important inhibitory receptors of NK cells, is negatively associated with their antitumor activity [73, 74]. Also, the cytolytic function of activated NK cells is largely depended on the release of granzyme B and perforin that mediate contact-dependent NK cells function [16, 75]. As a fact, tumor cells use different mechanisms to manipulate the expression of these molecules on NK cells, shifting the balance toward tumor progression [16]. Because TDEs mirror the content of parental cells, thus it is highly likely that these particles also play a part in altered receptor and molecular expression, underpinning tumor-associated NK cells dysfunction [76]. Recently, investigating the effects of oral cancer-derived exosomes (OCEXs) on NKG2D, NKp30, NKp44, NKp46 and NKG2A expression by NK cells, researchers observed that OCEXs significantly upregulate the expression of activating receptors (NKG2D, NKp30, NKp44 and NKp46) on the NK92MI cells for 24 h following treatment, whereas the expression of NKG2A was remarkably decreased at the same timeline [22]. Notably, further experiments revealed a gradual decrease in the expression of activating receptors on NK cells over the time for 7 days, while, no significant changes were observed for NKG2A expression in this time [22]. Since tumors constantly release exosomes into surrounding microenvironment as well as into the circulation, thus NK cells are more likely to be continually exposed to tumor exosomes, leading to an ultimate loss of cytotoxic function in NK cells. Others have also shown that murine mammary carcinoma (TS/A cells) exosomes selectively modulate the expression of cytolytic effector molecules in NK cells [35]. It was found that co-culture of the cytokine activated-NK cells with tumor exosomes dramatically reduce perforin in a dose-dependent manner, while the expression of granzyme B did not change [35]. Interestingly, pretreatment of NK cells with murine breast cancer exosomes was observed to not affect the expression of perforin mRNA, indicating that tumor exosomes might affect perforin expression at protein levels [35]. Exosomes derived from multiple myeloma and pancreatic cancer cells (L3.6pl cells) were also demonstrated to downregulate the expression of NKG2D on natural killer (NK) [34, 55]. Furthermore, saliva exosomes from pancreatic ductal carcinoma (PADC) bearing mice were also demonstrated to trigger surface NKG2D down-modulation and significantly decrease granzyme B and perforin expression [60]. The same results were also reported by others. It has been shown that CD34 + exosomes from AML patients' plasma (circulating blast-derived exosomes) significantly inhibited NKG2D expression on NK cells and reduced NKp46 [77]. Mesothelioma-derived exosomes co-cultured with peripheral blood lymphocytes (PBLs) were also shown to reduce both the proportion of NKG2D-positive cells as well as the surface expression of NKG2D [78]. This phenotypic alteration was specific, as it was noted that tumor-exosome treatment did not alter expression of CD3, CD4, CD8, CD56, or CD16 [78]. In particular, the expression level of CD94, a molecule that is also expressed in association with NKG2 receptors on a subset of NK cells and CD8 T cells, was found unchanged following tumor exosome treatment [78]. To determine whether TDEs-mediated NKG2D down-modulation can affect NK cell activation, researchers have examined the expression of the activation marker CD69. As a result, treatment with tumor exosomes was observed does not alter CD69 expression [78]. Similarly, no changes were observed in the constitutive perforin or granzyme B expression. These findings implied that tumor exosomes mediate these effects directly, and do not require the activity of CD4 T cells, or dendritic cells [78]. Although surface NKG2D was decreased following exosome treatment, however the total cellular NKG2D was largely stable, confirming that tumor exosomes induce internalization of NKG2D from the surface [78]. This provides the evidence that tumor exosomes can selectively trigger a decrease in cell surface NKG2D, without concomitant cellular activation. Consistently, NK and CD8 + T cells isolated from patients with castration-resistant PC (CRPC) exhibit a significant decrease in surface NKG2D expression compared to healthy individuals [79]. Co-incubation of exosomes isolated from serum or plasma of CRPC patients with lymphocytes from healthy donors also triggered the downregulation of NKG2D expression. To further clarify these observations, researchers have examined the expression of NKG2D by PBMCs after in vitro incubation with prostate cancer 22Rv1 cells-derived exosomes [79]. Significantly reduced cell-surface NKG2D expression was observed after 24 h in CD8 + T cells and NK cells treated with 22Rv1 exosomes [79]. This downregulation was evident both as a decrease in the proportion of NKG2D-positive cells and a reduction in the mean fluorescence intensity (MFI) compared to untreated PBMCs. It was concluded that this reduced NKG2D expression induced by 22Rv1 exosomes was NKG2D receptor-specific, since exosome treatment did not change the expression of CD3, CD8, CD56, CD16 or the activation marker CD69 [79]. Further experiments were also done to test whether the NKG2D receptor was internalized or merely masked on the cell surface by exosomes. The findings showed that the cell-surface downregulation of NKG2D by 22Rv1 exosomes was dose-dependent and mediated by the receptor internalization. More importantly, 22Rv1-derived exosomes were able to downregulate the NKG2D-dependent killing ability of PBMCs from healthy donors [79]. Together, these results support the notion that TDEs may affect NK cells function through downregulating NKG2D, granzyme and perforin expression (Fig. 1f).

### What molecules underlie TDEs-mediated NK cell dysfunction?

Tumor exosomes can actively induce immunosuppression through several mechanisms and cellular or subcellular pathways [12, 80]. A variety of immunoinhibitory proteins on their surface, as well as lipid and RNA (miRNA, lncRNA, etc.) content of TDEs can influence immune cells, including NK cells [12]. Here, we summarized the previously evidenced biomolecules that are involved in TDEs-mediated NK cells dysfunction (Table 1).

**Table 1:** The bimolecular cargo of TDEs and their inhibitory effects on NK cells

Exosomal Cargo	Cell of Origin	Mechanism of Action	Ref
**Ligands for NKG2D (MICA/B and ULBP1-6)**			
	AML	Downregulate NKG2D expression and reduce NK-cell cytotoxicity	[23]
	AML	CD34 + exosomes downregulate NK cells activity through decreasing NKG2D levels	[77]
	Metastatic melanoma	Downregulating NKG2D expression on NK cells	[81]
	Saliva exosomes (pancreatic ductal carcinoma (PADC)	Decreases NK cell activation level and triggers downregulation of surface NKG2D	[60]
	HELA, HepG2 and MelJuso	Particle-associated MICA (MICA*008) downregulates NKG2D expression	[82]
	Mesothelioma cell line, prostate cell lines (PC3 or DU145), and EBV-B lymphoblastoid cells (IB4)	Downregulation of surface MICA expression on NK cells	[78]
	Jurkat and Raji cell lines	Exosomal ligands for MICA/B and ULBP1 and 2 downregulate the expression of MICA/B	[83]
	22Rv1( human prostate carcinoma epithelial cell line)	Exosomal MICA/B and ULBPs downregulate NKG2D expression	[79]
	Epithelial ovarian cancer (EOC)	Induce NKG2D downregulation, but do not affect DNAM-1-PVR/nectin-2 pathway	[84]
**TGF-b**			
	AML	Decreases NKG2D expression through SMAD2/3 pathways in NK-92 cells, but do not affect DAP-10 expression	[23]
	AML	Down-regulation of NKG2D receptors and suppression of NK cells activity through the phosphorylation of SMAD1/5/8	[85]
	ALL	Induction of TGF-b signaling by upregulating MDS1 and EVI1 expression	[36]
	Pancreatic cancer	Delivering TGF-b to NK cells and activating the phosphorylation of Smad2/3 signaling pathway	[34]
	Oral cancer	Decreasing the expression of NKp30 and NKG2D on NK cells	[22]
	Non-small cell lung cancer (NSCLC)	Hypoxic MV had higher TGF-b levels and decreased surface NKG2D expression	[37]
	Clear cell renal cell carcinoma (ccRCC)	Abrogating cytotoxic function of NK cells through the activation of the TGF-b/SMAD signaling pathway	[64]
**Adenosine and Glucose Metabolism**			
	AML	Induces adenosine production in TME by expressing CD39/CD73 pair and impair NK cells function via A2AR	[23]
	Glioblastoma	Carrying CD39 and CD73 and mediate NK cells dysfunction	[63]
	Pancreatic cancer cells (L3.6pl)	Reduces the expression of CD71 (transferrin receptor), CD98 (large neutral amino acid transporter) on NK cells	[34]
**Fas-L, Survivin, B7-H3 and PD-L1**			
	Lymphoma	Exosomal Fas-L and Survivin induces NK cell impairment by reducing the expression of perforin, granzyme B, TNF-α, IFN-γ and NKG2D	[86]
	Glioblastoma cells	B7-H3 carrying exosomes impair NK-mediated tumor lysis	[87]
	Melanoma	PD-L1 + exosomes induce NK cells dysfunction through PD-L1/PD1 axis	[88]
**RNAs**			
	Hepatoma	miR-92b containing exosomes alter CD69 expression on NK cells and impair their activity	[89]
	NSCLC	Downregulating CD107a through miR-23a	[37]
	Hepatocellular carcinoma (HCC)	-Ring finger domain 1 RNA (circUHRF1) containing exosomes decrease proportion and infiltration of NK cell -Exosomal circUHRF1 enhancing TIM-3 expression via degradation of miR-449c-5p	[90]
	Colorectal cancer	lncRNA SNHG10 induces inhibin subunit beta C (INHBC), which is involved in the TGF-β signaling pathway	[91]

### Exosomal ligands for NKG2D (MICA/B and ULBP1-6)

**Fig. 2:**
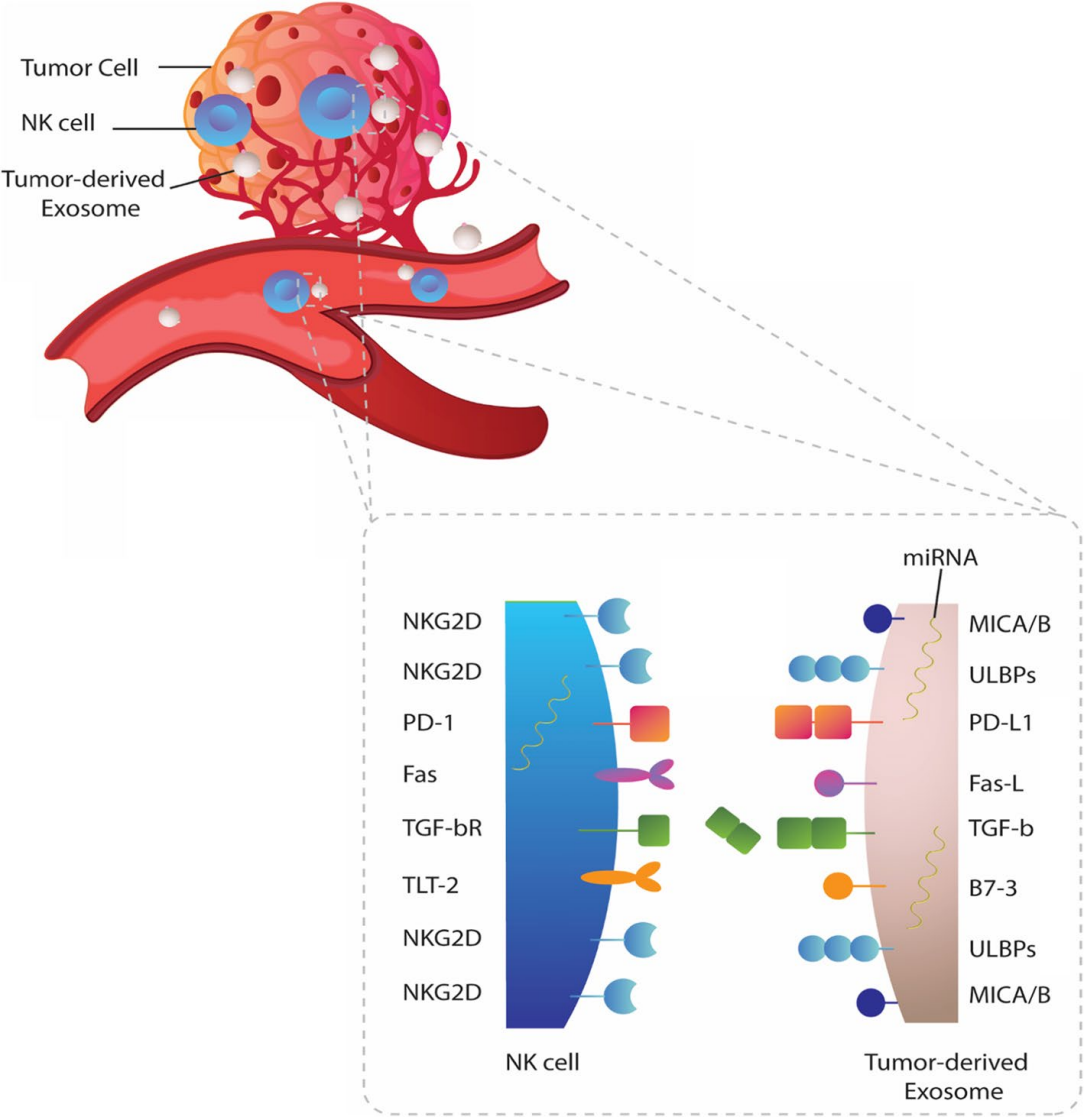
The immunological synapse between tumor-derived exosomes and NK cells. Tumor cells release a large amount of immunoinhibitory exosomes into tumor microenvironment and circulation which can interact with NK cells and deliver their suppressive content into these cells. A plethora of biomolecules, including MICA/B, ULBPs, PD-L1, Fas-L, TGF-b, and B7-3 presents on exosomes that can be engaged with their cognate receptors on NK cells and induce downstream signaling, inhibiting their anti-tumor activity

The continuous exposure of NK cells to ligands expressed on the surface of tumor cells have been demonstrated to result in NK cells abnormalities [92, 93]. As mentioned earlier, the absence or downregulated levels of NKG2D are a common feature of functionally suppressed NK cells that might be induced by tumors or soluble factors derived from tumor/surrounding cells [94, 95]. Studies show that the MHC class I-related chain (MIC) A and MICB ligands for the activating receptor NKG2D can be shed from tumor cells and their presence in patients’ plasma is closely associated with the compromised NK cell responses and disease progression [96, 97]. Because the presence of MICA and MICB on tumor exosomes has been validated, thus it is likely that exosomal MICA/B might be able to alter NKG2D expression similar to their soluble counterparts [98–100]. Concerning this, AML exosomes were revealed to be rich in membrane-associated MICA/MICB that are assumed to be responsible for NKG2D downregulation and concomitant reduction of NK-cell cytotoxicity [23]. Others have also reported shedding of the most frequently expressed MICA allele in human populations (MICA*008) into exosomes [82]. Based on the findings, NK cells treated with MICA*008 containing exosomes derived from human cervical cancer Hela cells had significantly downregulated expression of NKG2D with a marked reduction in cytotoxic activity. Comparing the effect of MICA*008 (released in exosomes) or MICA*019 (soluble) on the expression of NKG2D on primary human NK cells, it was found that culture supernatants containing either MICA*019 or MICA*008 can significantly decrease cell surface NKG2D expression, whereas MICA*008 containing supernatant (exosomal form) induced more downregulation of NKG2D [82]. Likewise, examining whether the down-modulation of NKG2D was dependent on exosome phenotype, a previous study found that exosomes isolated from NKG2D ligand-positive tumor cells, including mesothelioma cell line, prostate cell lines (PC3 or DU145), EBV-B lymphoblastoid cells (IB4), or exosomes purified from a mesothelioma patient’s pleural fluid (PF) were capable of driving down the expression of NKG2D in NK cells [78]. In contrast, exosomes from PBMCs or fibroblasts had no effect on NKG2D expression. These findings showed that the decrease in NK cell NKG2D is exosome phenotype dependent, and occurs with tumor but not with non-tumor exosomes [78]. To determine whether exosomal NKG2D-ligand expression is involved in this down-regulation, similar assays were performed using mesothelioma cell line-derived exosomes, that strongly express surface MICA, in the presence of neutralizing MICA-specific Ab or isotype-matched control Ab [78]. It was observed that the exosome-mediated down-modulation of NKG2D was inhibited by anti-MICA, but not isotype- control Ab, confirming that reduced NKG2D surface expression occurs with tumor but not with non-tumor exosomes, and is at least in part due to exosomal MICA expression [78]. In a similar setting of experiments, pre-incubating with anti-ULBP and anti-MIC mAbs significantly inhibited the downregulation of NKG2D expression induced by exosomes from prostate cancer cells (22Rv1 cells) [79]. However, on the other hand, pre-treatment of 22Rv1 exosomes with an anti-CD63 mAb, a known exosomal protein, did not significantly affect the expression of NKG2D [79]. The immunosuppressive ability of epithelial ovarian cancer (EOC) exosomes on two cytotoxic pathways of importance for anticancer immunity, the NKG2D receptor-ligand pathway and the DNAM-1-PVR/nectin-2 pathway, has also been investigated [84]. It was shown that exosomes isolated from EOC tumor explant and EOC cell-line culture supernatants, and ascitic fluid from EOC patients differentially and constitutively express NKG2D ligands from both MICA/B and ULBP families on their surface, while DNAM-1 ligands are more seldom expressed and not associated with the exosomal membrane surface [84]. The NKG2D ligand-bearing EOC exosomes was found to significantly downregulate the NKG2D receptor expression on peripheral blood mononuclear cells (PBMC) while the DNAM-1 receptor was remained unaffected [84]. Of note, the downregulation of NKG2D expression was observed to be associated with lesser NKG2D receptor-ligand-mediated degranulation and cytotoxicity of NK cells in vitro against OVCAR-3 and K562 cells. It was inferred that the EOC exosomes act as a decoy, thereby impairing the NKG2D-mediated activity of NK cells [84] (Fig. 2). Furthermore, investigating whether TDEs contribute to the immune evasion from NK surveillance as a cause of high relapse and fatal outcome of many blood malignancies [83, 101], researchers have employed Jurkat and Raji cell lines, as a model for studies of the NKG2D receptor-ligand system in T-and B cell leukemia/lymphoma [83]. Preliminary findings showed that Jurkat and Raji cells constitutively express mRNA and protein for the stress-inducible NKG2D ligands MICA/B and ULBP1 and 2, and actively release them by exosomes. Further results showed that the NKG2D ligand-bearing exosomes derived from Jurkat and Raji cell lines could act as a decoy and downregulate the NKG2D receptor-mediated cytotoxicity of NK cells in vitro [83]. Emphasizing the role of exosomal ULBPs, others have also revealed that tumor cells release exosomal ULBP3 which is more potent for NKG2D downregulation compared to soluble ULBP2, limiting cytotoxic activity of NK cells [102]. These results might partly explain the clinically observed NK-cell dysfunction in patients suffering from leukemia/lymphoma, and suggest that exosomal ligands for NKG2D play crucial roles in tumor-associated NK cells abnormalities. As a matter of importance, thermal and oxidative stress was found to enhance the secretion of NKG2DL-bearing exosomes that aggravated the impairment of the cytotoxic response by NK cells [83]. Therefore, the adverse effect of thermal and oxidative stress, enhancing the release of immunosuppressive exosomes, should be considered when cytostatic and hyperthermal anti-cancer therapies are designed [83].

In spite of carrying exosomal NKG2D ligands on their surface, it is strongly speculated that cancer-derived exosomes may also influence cellular function through a variety of biological mechanisms to the benefit of the tumors that produce them [103, 104]. For example, due to its expression on exosomes, previous studies inferred that exosomal MICA derived from metastatic melanoma cell line (Ma- Mel-86c) is responsible for the NKG2D downregulation [81, 105, 106]. On the contrary, a recent study on exosomes from melanoma patients revealed that although melanoma exosomes could induce NKG2D downregulation on NK cells, but this effect is unrelated to exosomal MICA/B [88]. These results support that exosomal NKG2D ligands are, in part, responsible for downregulating the expression of NKG2D receptor, and there might be other exosomal biomolecules rather than MICA/B that are involved in this phenomenon (Fig. 2).

### Exosomal TGF-b

**Fig. 3:**
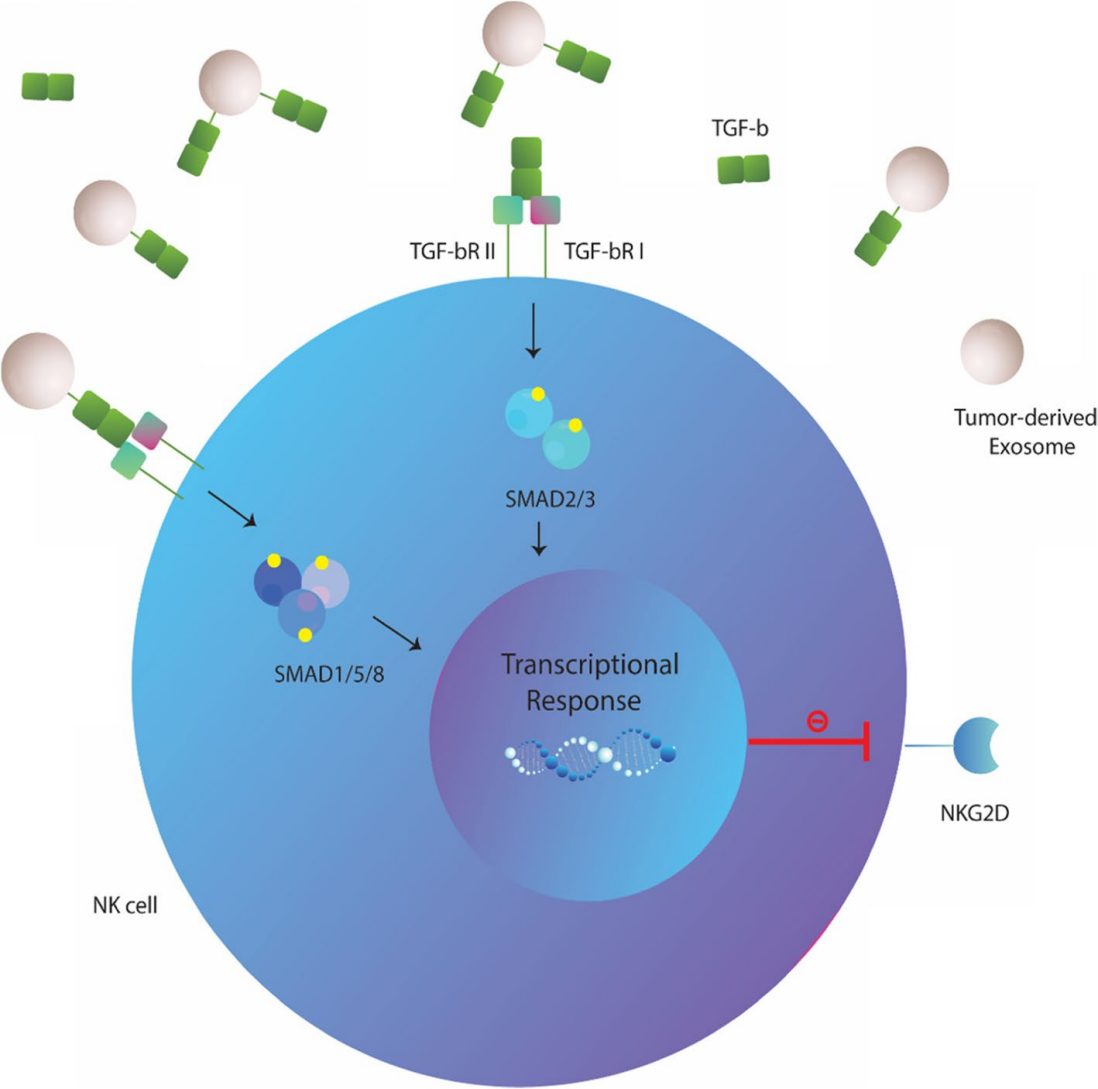
Exosomal transforming growth factor-beta (TGF-b) induces NK cells dysfunction. Tumor-derived exosomes (TDEs) harbor higher levels of membrane-associated mature TGF-b that can be dissociated to increase TGF-b levels in the tumor microenvironment. Both membrane-bound and soluble forms of TGF-b (released from tumor exosomes) are capable of binding to TGF-bRI/TGF-bRII on NK cells that result in SMAD2/3 or SMAD1/5/8 phosphorylation, which are subsequently translated into the lower expression of NKG2D and associated activation of NK cells

Transforming growth factor-beta (TGF-b), a cytokine of the bone morphogenetic protein (BMP)-activin family, is well-known to participate in a wide range of processes involving regulation of immune responses [107–109]. It is especially acknowledged for its inhibitory effects on immune cells, including NK cells [110, 111]. Strong evidence show that TGF-b reduces the surface expression of crucial activating receptors (NKp30 and NKG2D) on NK cells and elevated levels of this cytokine is associated with impaired activity of NK cells in cancer patients [112–115]. Most recent studies indicate that tumor exosomes also carry high levels of membrane-associated TGF-b that may induce immunosuppressive effects similar to its soluble counterpart. There are now multiple reports showing that exosomes/MVs isolated from AML and clear cell renal cell carcinoma (ccRCC) patients or mesothelioma cells contain significant levels of mature TGF-b [23, 78, 85, 116]. Nevertheless, a pro-peptide isoform of TGF-b comprised of the LAP covalently bound to mature TGF-b (~ 50 kDa) has also been reported on AML exosomes (99), and this latent form of TGF-b was shown to be dissociated following treatment with urea, further increases soluble TGF-b levels [85]. Of particular note, there is a broad consensus that TGF-b containing tumor exosomes are capable of driving down NKG2D levels on NK cells comparable to that seen for soluble TGF-b, whereas neutralizing antibodies against TGF-b can restore the observed effects [23, 64, 78, 85, 116]. In addition, slight or no further changes were seen for the NKG2D expression, when recombinant TGF-b was added to tumor exosomes, indicating that tumor exosomes alone can maximally activate the TGF1-dependent NKG2D down-regulation pathway [23, 78]. Based on these observations, researchers have assumed that exosomal TGFβ may interact with TGFβRI/II receptors on the cell surface, inducing inhibitory downstream signals in NK cells that leads to the decreased NKG2D expression [23, 64]. This becomes evident as SMAD2/3 phosphorylation was observed to be increased in NK cells co-incubated with TGFβ + exosomes [23, 64]. TGF-b containing exosomes were also shown to downregulate the expression of Tbet transcription factor in NK cells [23]. However, TGFβRI/II inhibitor (LY2109761) or TGF-b knockdown could decrease SMAD 2/3 phosphorylation to the baseline levels [23, 64]. These findings strongly support that tumor exosomes can deliver TGF-b to the surface of NK cells that is engaged with TGFβRI/II and subsequently upregulates SMAD 2/3 phosphorylation and decreases Tbet expression levels, leading to the decreased NKG2D expression and abrogated cytolytic activity in NK cells [23, 64] (Fig. 3). Likewise, others have shown that TGF-b can be transferred to NK cells by oral cancer-derived exosomes (OCEXs) and extracellular vehicles (EVs)-derived from pancreatic cancer that activates the phosphorylation of Smad2/3 signaling pathway, ultimately resulting in NK cell dysfunction [22, 34]. Besides, some experiments showed that exosomal TGF-b induces the phosphorylation of SMAD1/5/8 in NK cells, resulting in lower expression of NKG2D, where the addition of anti-TGF-b antibodies restored these effects [85] (Fig. 3). A recent study also found that acute lymphoblastic leukemia (ALL)-derived exosomes alter NK92-MI cells function mainly through signaling of the TGF-b pathway [36]. Observations revealed that ALL exosomes upregulate the expression of several genes related to the TGF-b signaling pathway, including MDS1 and EVI1, that enhances TGF-b signaling and its inhibitory effects [36, 117]. Taken together, all these data suggest a central role for exosomal TGF-b in decreasing NKG2D expression and tumor–induced NK cells dysfunction.

Worth noting, exosomes derived from T (tumor) region had higher TGF-b than that from NT (non-tumor) region [64]. On the other hand, TDEs isolated before, during and after chemotherapy exhibited different levels of active TGF-b (99), and only exosomes containing high levels of mature TGF-b were able to inhibit expression of NKG2D in purified normal human NK cells [64, 95]. Such a data implies that the inhibitory effects of TGF-b + exosomes are more pronounced at tumor site and on tumor-infiltrating NK cells rather than circulating NK cells.

Furthermore, since hypoxic tumor microenvironment has been verified to modulate TGF-b signaling and exosomal cargo loading, researchers have also examined whether hypoxia can affect expression of NK ligands and TGF-b on non-small cell lung cancer (NSCLC)-derived MVs [37]. Although, no significant changes were observed in expression of NK ligands on hypoxic TD-MVs, but interestingly, MVs isolated from hypoxic cells (hypoxic IGR-Heu and K562 tumor cells) had significantly higher levels of TGF-b compared to those from normoxic tumor cells [37]. Of particular note, treatment of NK-92 and NKD cells with MVs of hypoxic tumor cells dramatically decreased surface NKG2D expression, which was abolished by anti-TGF-b blocking antibody [37]. Targeting vesicular TGF-b also restored the IFNγ production by NK-92 and NKD cells. These results were consistent with the observations that hypoxic derived MVs had remarkably greater inhibitory effect on NKG2D expression in comparison to normoxic MVs [37]. Overall, it was concluded that hypoxia suppresses NK cells activity, in part, by inducing TGF-b sorting into MVs released by tumors, while the levels of particle-associated NKG2D ligands are somehow stable. This is somehow in line with the previous findings showed that exosomes isolated from tumors of higher stages exhibited more immunosuppressive cargo than those from early stages [37, 118]. Because hypoxia is a more common feature of advanced stage tumors, thus higher levels of TGF-b would be expected to present on TDEs with the tumor progression. Accordingly, it can be postulated that tumor progression (hypoxia condition) can strengthen the effects of exosomal TGF-b on NKG2D downregulation and tumor-associated NK cells dysfunction.

### Exosomal adenosine and glucose metabolism

Adenosine (ADO) is a well-recognized inhibitor of immune cell functions [119–121]. As a part of fact, adenosine can interact with A2AR receptors on immune cells, inducing a cascade of downstream signals that upregulates cAMP and inhibits cellular function [120, 122–124]. Studies have shown that tumor exosomes carry CD39/CD73 actively contribute to the suppression of anti-tumor T cells and the production of ADO by Treg cells [125]. In the same direction, NK-92 cells were observed to produce significant levels of ADO, inosine and hypoxanthine in the presence of exogenous ATP and CD39 + /CD73 + AML exosomes [23]. It was noted that AML exosomes are able to convert exogenous ATP into ADO, however, although NK-92 cells express CD39/CD73 pair and A2AR, but they do not produce ADO in the presence of exogenous ATP. Strikingly, in the presence of tumor exosomes, NK cells are able to produce ADO from exogenous ATP. Therefore, it can be inferred that exposure to exosomal CD39/CD73 forces NK cells to produce ADO, and because NK-92 cells carry A2ARs, it is very likely that autocrine signaling of ADO or inosine binding to the A2ARs expressed on NK-92 cells may, in part, responsible for a loss of function in NK-92 cells [126] (Fig. 1h). Glioblastoma-derived exosomes (GBex) have also been shown to carry CD39 and CD73, and ADO produced by these exosomal proteins is assumed to be partly responsible for GBex-mediated NK cell dysfunction [63]. In spite of adenosine, nutrient uptake and glucose metabolism are also important for proper NK cell responses [127, 128]. CD71 (transferrin receptor), CD98 (large neutral amino acid transporter), and 2-NBDG incorporation ability are three commonly-used metabolic parameters in NK cells [34]. However, pancreatic cancer cells (L3.6pl)-derived EVs were shown to significantly reduce the expression of CD71 and CD98 in NK cells and their glucose uptake capability [34]. Such findings underscore the importance of TDEs in mediating metabolic reprograming of NK cells (Fig. 1h).

### Exosomal fas-l, survivin, B7-H3 and PD-L1

Increasing evidence shows that TDEs harbor a plethora of membrane-associated proteins that are crucially participated in tumor-immune escape [42, 129]. Several studies have demonstrated that TDEs carry Fas-L can induce apoptosis in TCD8 + cells [130–135]. Others have also shown that tumor cells actively release Fas-L bearing EVs to kill Fas-expressing cells in TME, including NK cells [63]. In a research on lymphoma, it was found that lymphoma-derived exosomes are enriched in Fas-L and Survivin, a member of the inhibitor of apoptosis (IAP) proteins, but did not contain MICA/B and TGF-b [86]. Findings revealed that treatment with the lymphoma-derived exosomes downregulate the expression of NKG2D on NK cells and decrease protein levels of perforin, granzyme B, TNF-α, and IFN-γ which might be Fas-L/Survivin dependent [86].

B7-H3, a member of the B7-family proteins, is another checkpoint molecule has been shown to enforce immunosuppression in a variety to tumors [136–138]. Previously, it was found that glioblastoma cells secret B7-H3 in exosomal manner that can suppress NK-mediated tumor lysis [87]. Additionally, the majority of most recent studies investigating the roles of TDEs in cancer biology pointed out that exosomes also play a major role in tumor-immune escape through PD-L1/PD1 axis [51, 139–141]. It was demonstrated that TDEs carry functional isoform of PD-L1 that robustly act against antitumor immunity [33, 51]. Furthermore, it has been shown that PD-L1 positive exosomes counteract the benefits of anti-PD-1/PD-L1 mAb therapy [139, 142]. Since activated NK cells also express PD-1, it is thus conceivable that PD-L1 bearing tumor exosomes exert inhibitory effect on NK cells [143]. In this regard, a recent study showed that melanoma-derived exosomes induce NK cell dysfunction partly through PD-L1 expression [88]. However, controversially, PD-L1 + exosomes from plasma of AML patients have been suggested to not contribute to the suppression of NK-92 activity [23]. Overall, these findings support the view that tumor exosomes carry various biologically-active molecules with the potential to activate inhibitory molecular pathways in recipient cells, including NK cells (Fig. 2).

### Exosomal RNAs

In addition to proteins, TDEs contain mRNAs, microRNAs and lncRNAs that can be taken-up by other cells, including NK cells and alter their function [144–147]. Investigating the effects of circulating exosomes on hepatocellular carcinoma (HCC) development and recurrence after living donor liver transplantation (LDLT), it was found that hepatoma-derived exosomes contain high levels of miR-92b [89]. Experimental results revealed that exosomes derived from miR-92b overexpressing Hep3B cells are also rich in miR-92b. In particular, treatment with miR-92b containing exosomes remarkably induced miR-92b expression in NK-92 cells, indicating that tumors actively transfer inhibitory biomolecules, including miRNAs, to infiltrating NK cells via exosomes [89]. Because of its pivotal role in NK cells function, researchers have examined whether exosomal miR-92b can alter CD69 expression on NK cells. Subsequent results showed that transfer of miR-92b via TDEs could significantly inhibit CD69 expression on NK-92 cells and reduced their cytotoxicity against parental Hep3B tumor cells. Furthermore, the overexpression of miR-92b was found to be associated with the enhanced migration of liver cancer cell lines [89]. The same results were also repeated by primary NK cells from mouse and mouse lymphoma YAC-1 cells. These results demonstrate that tumor-derived circulating exosomes are able to transfer their miRNA content into tumor-infiltrating immune cells, including NK cells, and thereby suppress their anti-tumor activities in favor of tumor progression. In another research on NSCLC, the role of hypoxic tumor-derived MVs in transferring miRNAs to mediate NK cell dysfunction has been investigated [37]. Profiling MVs from both normoxic and hypoxic tumor cells revealed the presence of miRNAs. Of particular note, hypoxic MVs were found to have higher amounts of miR-210 and miR-23a compared to normoxic MVs. Further findings showed that hypoxic MVs containing miR-23a could impair NK cells cytolytic activity by downregulating CD107a, while miRNA-210 did not affect NK cells function [37]. Moreover, miR-23a containing exosomes was observed to not alter the receptor expression patterns as well as the expression levels of IFNγ and granzyme B in NK cells, suggesting that its ability to suppress NK cells cytotoxicity is most likely related to its effect on CD107a expression. This was confirmed as the hypoxic MVs-mediated decrease in the percentage of CD107a and IFNγ-positive NK cells was reversed when MVs were transfected with pre-miR-23a [37] (Fig. 1g).

Others have also shown that HCC exosomes contain high levels of ubiquitin-like with PHD and ring finger domain 1 RNA (circUHRF1) comparable to that seen in human HCC tissues [90]. It has been demonstrated that circUHRF1 is secreted in an exosomal manner into plasma of HCC patients mainly by tumor cells. Moreover, circUHRF1 was found to inhibit IFN-γ and TNF-α secretion by NK cells and high levels of plasma exosomal circUHRF1 is closely associated with a decreased proportion of NK cell and their decreased infiltration into tumor microenvironment [90]. Furthermore, circUHRF1 was shown to drive resistance to anti-PD1 therapy in HCC patients and inhibits NK cells function by enhancing TIM-3 expression via degradation of miR-449c-5p [90]. A most recent study also showed that exosomal lncRNAs play major roles in exosome-mediated immune escape of colorectal cancer (CRC) from killing by NK cells [91]. Based on the results, exosomes derived from an epithelial-mesenchymal transition (EMT) model of SW480 cells were able to suppress the proliferation, cytotoxicity, production of IFN-g and secretion of the perforin and granzyme B of NK cells [91]. Further surveys showed that these exosomes contain lncRNA-SNHG10 that mediates the decreased viability and suppression of NK cells [91]. Transcriptome sequencing showed an upregulation of 114 genes in NK cells treated with exosomes containing lncRNA-SNHG10, including inhibin subunit beta C (INHBC), which is involved in the TGF-b signaling pathway. Putting these findings together, it can be postulated that TDEs act as a major mediator of tumor-associated NK cells dysfunction, that partly rest on their ability to transfer RNAs (Fig. 1,1g).

### Clinical significance and therapeutic approaches

**Fig. 4:**
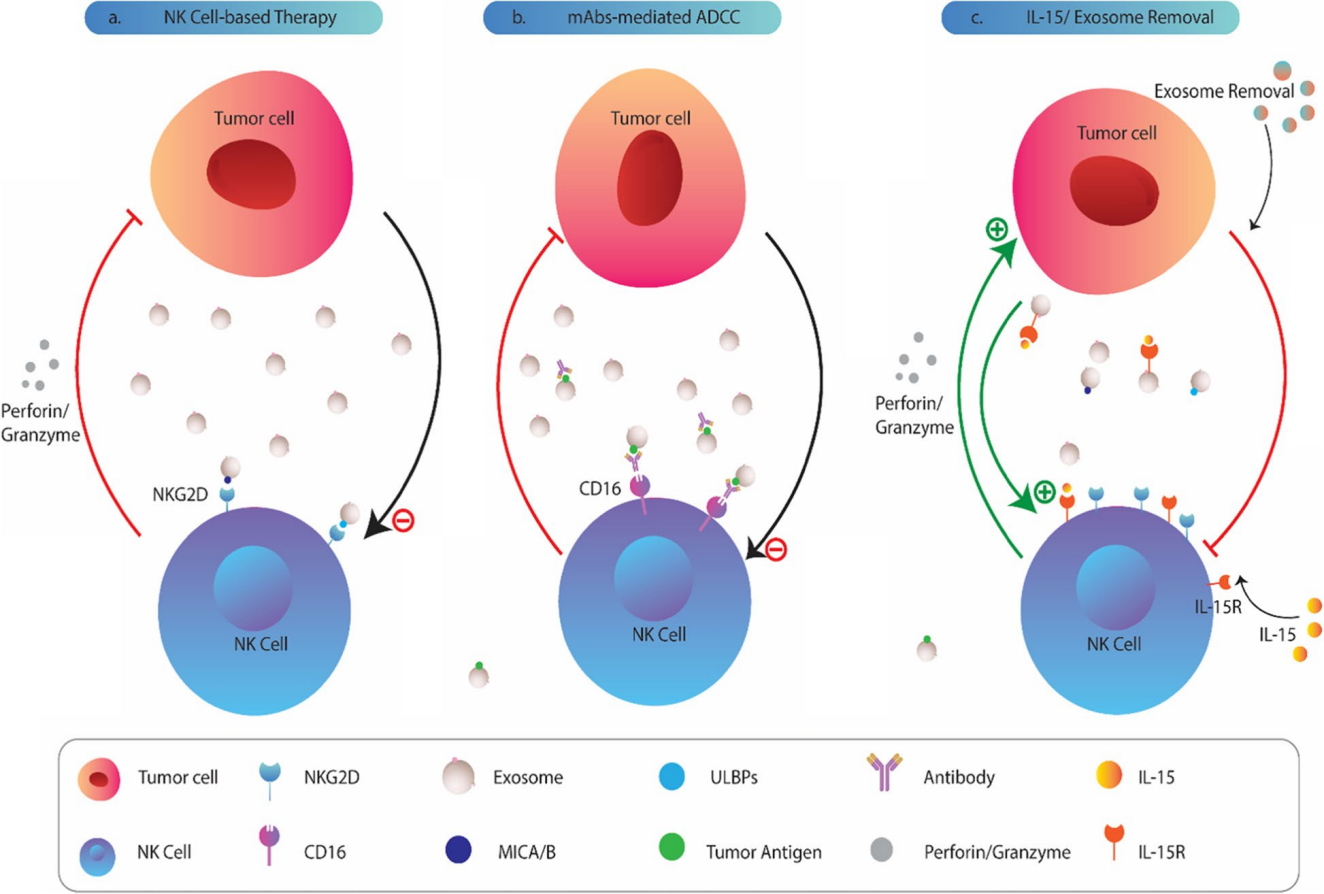
Clinical significance of tumor exosomes in NK cell-based therapies and potential therapeutic approaches. **a**) Tumor exosomes serve as decoy molecules that interact with NKG2D receptors and decrease the cytolytic activity (secretion of perforin and granzyme) of NK-92 cells in treating AML. **b)** Exosomes also harbor tumor associated antigens (TAAs) that can bound to therapeutic monoclonal antibodies and interfere with the NK cell-mediated antibody dependent cell cytotoxicity (ADCC), decreasing therapy efficacy. **c)** Removing cancer exosomes from tumor microenvironment or adding IL-15 could restore cytolytic activity of NK cells and serve as adjunctive therapy for cancer treatment. Tumor exosomes also express IL-15R that can trans-present IL-15 to NK cells and further improve their anti-tumor function

NK cells are increasingly proven as a promising tool for cancer treatment, especially blood malignancies [17, 148, 149]. There are also great numbers of ongoing clinical trials investigating the efficacy of NK cell immunotherapy in treating solid tumors [69, 150, 151]. However, recently in a study on AML patients, it was revealed that circulating exosomes derived from tumor cells counteract the beneficial effects of adoptive NK-92 cell therapy [23] (Fig. 4a). As a fact, NK cells are also involved in anti-tumor immunity by a well-known antibody-dependent cellular cytotoxic (ADCC) process [152–154]. However, strikingly, previous studies have shown that tumor exosomes abolish the ADCC activity of NK cells [155]. For example, it has been shown that HER2 expressing exosomes act as a decoy to protect cancer cells from ADCC-mediated by NK cells. Such findings support the notion that tumor exosomes crucially devastate the therapeutic effects of NK cell-based therapy and monoclonal antibodies (mAbs) [155] (Fig. 4b). On this basis, as reviewed elsewhere, great efforts have been made in recent years to inhibit/remove circulating tumor exosomes as adjunctive therapy for cancer [14, 156–158]. It seems that targeting exosomes can improve anti-tumor immune responses and therapeutic effects of currently available immunotherapies [14, 156] (Fig. 4d).

On the other hand, there are shreds of evidence indicating that adding IL-15 undoes the inhibitory effects of TDEs on NK cells. A previous study showed that addition of IL-15 to cocultures of MVs isolated from sera of AML patients with NK cells from normal donors significantly abrogated the MVs-mediated suppression of NK cell activity [85]. As mentioned previously, co-incubation of NK cells with MVs from AML patients up-regulates phospho-SMAD expression. However, the pre-treatment of NK cells with IL-15 followed by co-incubation with MVs derived from sera of AML patients prevented MVs-induced phosphorylation of the SMAD pathway [85]. Moreover, in the presence of IL-15, the expression level of NKG2D was shown to remain unchanged in NK cells treated with MVs [85]. Similarly, it has been shown that tumor exosomes could impair IL-15-mediated up-regulation of NKG2D [78]. To clarify these observations, researchers have treated healthy donor PBLs with various concentrations of IL-15 either in the presence or absence of tumor exosomes [78]. The findings showed that in the absence of IL-15, tumor exosomes induce a significant reduction in NKG2D expression, but adding IL-15 could abolish the effect of exosomes and restore NKG2D to the baseline levels [78]. Confirming the above results, interesting findings from another study showed that low doses of chemotherapeutic agents, such as melphalan, could induce multiple myeloma cells to trans-present IL-15 to NK cells in an exosomal manner [26]. Detailed experiments revealed that both IL-15R and IL15 present on exosomes from melphalan-treated MM cells, where exosomal IL-15R mediate trans-presentation of IL-15 and can induce NK cells activation and proliferation [26]. These results are a proof of concept that IL-15 reverses the inhibition of NKG2D expression-mediated by tumor exosomes and protects NK cells from inhibitory effects of exosome-associated TGF-b (Fig. 4d).

### Perspectives and concluding remarks

It is clear from the studies examining the effects of TDEs on NK cells thus far that, as it has been described for other anti-tumor immune cells, cancer exosomes are important players of immunosuppression in tumor bearing hosts. Furthermore, it is strongly believed that these virus-sized particles interfere with the immunotherapies and induce resistance to chemotherapies [159, 160]. The concept of exosome inhibition/removal has gained prominence but, as we have reviewed elsewhere, it is still far from the clinical practice [14, 157]. However, despite that, it seems that adding IL-15 to immunotherapies, including NK cell-based therapies, could add to their benefits, in part, through interfering with the dampening effects of TDEs [156]. This notion is supported by the several clinical trials indicating that combination of IL-15 with NK cells or other immunotherapies augments the therapy effects and could remarkably increase remission [161]. On the other hand, adding anti-TGF-b, to interfere with exosomal levels of this protein, would also enhance therapy efficiency [162]. This becomes evident, as it has been shown that inhibiting TGF-b could improve the therapeutic efficacy of adoptive NK cell therapy [162–164]. Engineering exosomes to express HSP-70, IL-2 and IL12 or surface anchorage of staphylococcus enterotoxin A (SEA) onto TDEs have also been shown to enhance the anti-tumor activity of NK cells [27, 165–167]. Some reports also show that exosomes from tumor cells under genotoxic stress and epigenetic drug treatment or post-irradiation exosomes could enhance NK cells anti-tumor responses [25, 26, 30, 168, 169]. Thus, it seems interesting to employ engineered exosomes with customized cargo or some anti-cancer drugs, as an adjunctive treatment, to improve NK cells cytolytic activity. On the other hand, in spite of several biomolecules discussed in this review, there might be also some other unknown/less studied exosomal molecules that are involved in NK cell dysfunction in tumors [1, 170–172]. Therefore, further research are warranted to study the molecular basis of TDEs-mediated NK cells impairments. In summary, with deep understanding of the pathological roles and mechanisms underlie the TDE-mediated NK cells dysfunction; we would be able to further improve the therapeutic potential of NK cells, as well as other immunotherapies, in treating cancers.

## Data Availability

Not applicable

## Abbreviations

TDEs: Tumor-derived exosomes
NK cell: Natural killer cell
lncRNAs: Long non-coding RNAs
miRNAs: MicroRNAs
DCs: Dendritic cells
CTL: Cytotoxic T lymphocyte
KIR: Killer inhibitory receptor
MHCI: Major histocompatibility complex I;
MVs: Microvesicles
CXCL4: Chemokine (C-X-C motif) ligand 4
CXCL7: Chemokine (C-X-C motif) ligand 7
AML: Acute myeloid leukemia
CCL5: C–C Motif Chemokine Ligand 5
CXCR3: C-X-C Motif Chemokine Receptor 3
IL-2: Interleukin 2
IL-2R: Interleukin 2 receptor
MM-EXs: Multiple myeloma-derived exosomes
EVs: Extracellular vesicles
CSC: Cancer stem cell
OCEXs: Oral cancer exosomes
TD-MVs: Tumor-derived microvesicles
HNC: Head and neck cancer
GBex: Glioblastoma exosome
ccRCC: Clear cell renal cell carcinoma
IFNγ: Interferon-gamma
mRNA: Messenger RNA
PBMCs: Peripheral blood mononuclear cells
CRPC: Castration-resistant prostate cancer
PC: Prostate cancer
MICA/B: MHC class I-related chain A/B
EBV-B: Epstein bar virus-B
ULBP: UL16 binding proteins
mAbs: Monoclonal antibodies
EOC: Epithelial ovarian cancer
TGF-b: Transforming growth factor-beta
TGF-bRI/II: Transforming growth factor beta receptor I/II
(NSCLC: Non-small cell lung cancer
ADO: Adenosine
A2ARs: Adenosine A2A receptors
Fas-L: Fas-ligand
PD-L1: Programmed death-ligand 1
IAP: Inhibitor of apoptosis
TCD8 +: CD8 + T lymphocyte
TNF-α: Tumor necrosis factor-alpha
circUHRF1: Ubiquitin-like with PHD and ring finger domain 1 RNA
HCC: Hepatocellular carcinoma
TIM-3: T cell immunoglobulin and mucin domain-containing protein 3
ADCC: Antibody-dependent cellular cytotoxicity
